# Chitosan as a promising materials for the construction of nanocarriers for diabetic retinopathy: an updated review

**DOI:** 10.1186/s13036-024-00414-7

**Published:** 2024-02-22

**Authors:** Yan Lv, Chenglei Zhai, Gang Sun, Yangfang He

**Affiliations:** 1 Department of Ophthalmology, Jilin Province FAW General Hospital, Changchun, 130011 China; 2 Department of Orthopaedics, Jilin Province FAW General Hospital, Changchun, 130011 China; 3 Department of General Surgery, Jilin Province FAW General Hospital, Changchun, 130011 China; 4 Department of Endocrinology, the Second Hospital of Jilin University, Changchun, 130000 China

**Keywords:** Diabetic Retinopathy, Retina, Controlled release, Polymers, Chitosan

## Abstract

Diabetic retinopathy (DR) is a condition that causes swelling of the blood vessels of the retina and leaks blood and fluids. It is the most severe form of diabetic eye disease. It causes vision loss in its advanced stage. Diabetic retinopathy is responsible for causing 26% of blindness. Very insufficient therapies are accessible for the treatment of DR. As compared to the conventional therapies, there should be enhanced research on the controlled release, shorter duration, and cost-effective therapy of diabetic retinopathy. The expansion of advanced nanocarriers-based drug delivery systems has been now employed to exploit as well as regulate the transport of many therapeutic agents to target sites via the increase in penetration or the extension of the duration of contact employing production by enclosing as well as distributing tiny molecules in nanostructured formulation. Various polymers have been utilized for the manufacturing of these nanostructured formulations. Chitosan possesses incredible biological and chemical properties, that have led to its extensive use in pharmaceutical and biomedical applications. Chitosan has been used in many studies because of its enhanced mucoadhesiveness and non-toxicity. Multiple studies have used chitosan as the best candidate for manufacturing nanocarriers and treating diabetic retinopathy. Numerous nanocarriers have been formulated by using chitosan such as nanostructured lipid carriers, solid lipid nanoparticles, liposomes, and dendrimers for treating diabetic retinopathy. This current review elaborates on the recent advancements of chitosan as a promising approach for the manufacturing of nanocarriers that can be used for treating diabetic retinopathy.

## Introduction

Hyperglycemia or diabetes is the most common cause of death (1.5 million fatalities per year) in today’s world. WHO defines diabetes as “a long-term condition which develops when the body cannot use the produced insulin properly or when the pancreas cannot produce enough insulin“ [1–3]. According to the International Diabetes Federation’s (IDF) 2014 global survey, 387 million people have diabetes, with the prevalence expected to rise in 2035 by 592 million [4]. Diabetes consequences include nephropathy, neuropathy, or diabetic retinopathy. Among them, diabetes-related retinopathy (DR) represents the most commonly occurring and specific microvascular consequences of diabetes [5]. It is a deadly condition of the retinal vasculature [6]. DR is the primary cause of visual impairment among individuals of working age in developed countries [7]; 75% of DR-related vision loss is attributed to diabetic macular edema (DME), while the majority of the remaining balance loss is caused by complications of proliferative diabetic retinopathy (PDR). As a result, DR has a chance to pose a substantial danger to healthcare systems nowadays, as well as a future problem. Given the anatomy and physiology of the barriers of the eye, treating and managing pathological retinal neovascularization in the back of the eye is a difficult task [8]. Moreover, apart from the individual and economic consequences for the patient, the social effect of diabetic retinopathy is significant. The healthcare expenses for individuals with diabetic retinopathy are about twice as high as those without the condition [9]. Therefore, DR has the potential to emerge as a substantial socioeconomic burden on healthcare systems currently and a future challenge. Consequently, any therapeutic intervention capable of impeding or obstructing the advancement of diabetic retinopathy would yield tremendous benefits for both affected individuals and society. Moreover, the treatment and management of pathologic ocular neovascularization in the posterior segment of the eye in DR is a challenging endeavor due to the anatomy and physiology of barriers in the eye [10]. Current treatment strategies for DR include laser photocoagulation, intravitreal antivascular endothelial growth factor (anti-VEGF) injection, intravitreal triamcinolone injection, and vitreoretinal surgical intervention as a last step option [11, 12]. Over the years, ocular photocoagulation using lasers continues to be a common procedure for patients having retinopathy with sight problems, although the treatment does not always entirely recover lost vision [13, 14]. Vitrectomy surgery may be required in rare cases with advanced diabetic retinopathy [15]. Vascular endothelial growth factor (VEGF) or anti-vascular growth factor medicines are used for treating DR. However, anti-VEGF medications have been shown to delay the development of diabetic retinopathy and reduce the vision loss risk, they are currently extremely costly, involve an intrusive procedure, and have systemic and ocular risks. Use of these injections can cause bad consequences such as endophthalmitis, cataracts, hemorrhage of the vitreous, and retinal detachment. Anti-VEGF agent systemic exposure has proven that systemic VEGF suppression is related to unfavorable outcomes such as stroke, heart attack and bleeding. Researchers created steroid intravitreal implants to reduce systemic drug exposure, however this is also an intrusive technique with risks such as increased intraocular pressure, glaucoma and cataracts [16, 17]. Inhibitors of aldose reductase (ARI), inhibitors of growth hormone, anti-inflammatory drugs, protein kinase C (PKC) inhibitors, antioxidant substances, carbonic anhydrase inhibitors, therapy with genes or combination therapy also studied for managing and treating diabetic retinopathy [18]. However, because of a lack of research on the safety and efficacy studies, only a few therapy methods are available for managing diabetic retinopathy [19]. Many studies have been conducted to create a non-invasive, cost-effective, prolonged release, and enhanced therapeutic effect [20]. Several preclinical and clinical investigations have recently revealed that topical (noninvasive mode) delivery of the medicine reaches the back portion of the eye with the use of a novel colloid system (nanotechnology) [21]. Controlled release, low penetration, shorter duration, lack of targeted efficiency, invasiveness, low bioavailability in the eye, and side effects are some of the drawbacks associated with traditional techniques [22].

Ocular delivery with the help of nanoparticles is employed to maximize and regulate the transport of ocular therapeutics to target sites by increasing penetration or extending the duration of contact using production by enclosing and distributing tiny molecules in the nanostructured formulation [23]. By using a nanosized method, the pharmaceuticals can penetrate the ocular barrier for improved therapeutic efficacy than the traditional system [24, 25]. Continuous drug release is required to maintain the therapeutic levels and achieve better results in the intraocular tissues, but the use of nanomedicine provides sustained delivery of the medicine into the intraocular tissue via prolonged penetration as well as retention time thus improving bioavailability [26]. The current review provides an insight of diabetic retinopathy, nanocarriers for treating diabetic retinopathy and also aims to provide recent advancements in chitosan based nanocarriers for treating diabetic retinopathy.

## Diabetic retinopathy

### Retina

The retina covers the inner surface of the eye and is the light-sensitive tissue. The function of the retina is to take light, turn it into neuron impulses, and deliver electric signals to the brain enabling vision [27]. It is involved in the visual process and is made up of neuronal cells as well as a physiological barrier known as the blood-brain barrier (BRB). The retinal pigment epithelium and blood vessels are the two major components of BRB [28]. This physiological barrier is made up of a single layer of endothelial cells which are tightly joined. Because these connections are impenetrable to tracers, a wide range of chemicals can impact the eye’s metabolism [29]. The outer part of the blood-brain barrier is maintained by the retinal pigment epithelium [30]. Tight connections of retinal endothelial cells form the inner BRB, which is covered by the pericytes and glial cells [31]. Pericytes provide stability and regulate the proliferation of endothelial cells [32]. Glial cells play an important part in the varied processes of vascular components of the retina and the maintenance of blood flow in the retina [33]. Astrocytes and muller cells are the two types of microglial cells in the retina. Muller cells help the retina operate normally by maintaining ions and pH balance and astrocytes regulate the formation of the vasculature of the retina [34].

### Diabetic retinopathy pathophysiology

**Fig. 1:**
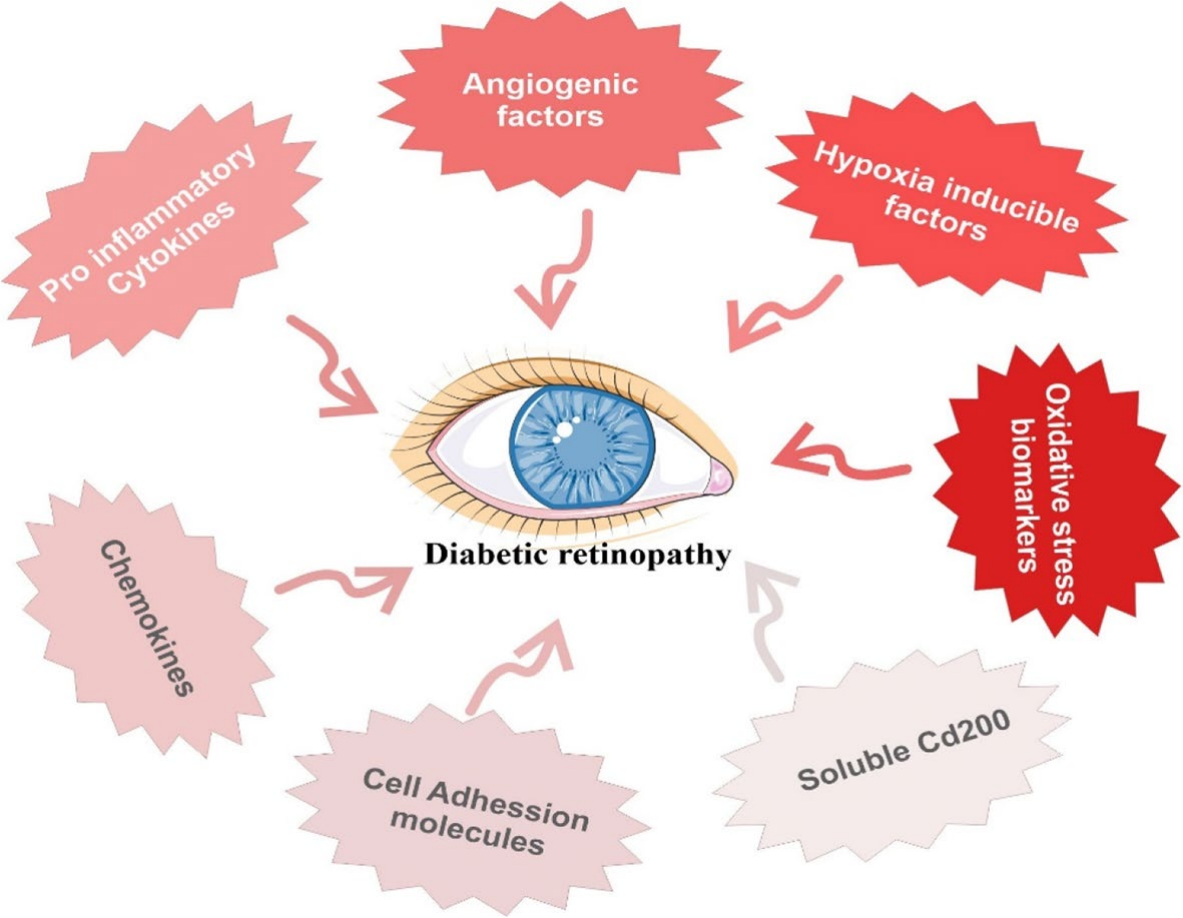
Factors inducing diabetic retinopathy

The most common cause is hyperglycemia [35]. The activation of chronic, poor-quality inflammatory signaling and metabolic dysfunction caused by high glucose plays a specific role in the course of DR [36]. Hemorrhages, microaneurysms, macular edema, capillary blockage, and, eventually, neovascularization are clinical symptoms of retinopathy [37]. DR pathogenesis involves several metabolic processes which include an increase in hexosamine pathway flux and activation of protein kinase C [38]. Furthermore, these events cause an increase in proangiogenic and inflammatory molecules such as VEG [39], angiopoietins [40], insulin-like growth factor [41], stroma-derived growth factor [42], interleukin-6 [43], hepatocyte growth factor (HGF) [43], and basic fibroblast growth factor [44]. Some of the most common factors that induce diabetic retinopathy have been shown in Fig. 1.

### Diabetic retinopathy classification

Diabetic retinopathy is categorized into two kinds based on disease severity: non- proliferative and proliferative diabetic retinopathy [45]. Non- proliferative diabetic retinopathy is classified as mild, moderate, and severe with the latter progressing to the most severe stage, proliferative DR, if not treated properly [46] and it is identified by observable retinal blood vessel injury. Blood arteries can acquire balloon-like swelling called microaneurysms at this early stage. It is the second stage of DR and during this stage, some of the tiny blood veins in the retina may get occluded [47, 48]. The obstruction of these tiny blood arteries reduces the flow of oxygen and nutrients to certain parts of the retina. Severe non-proliferative retinopathy is characterized by a considerable number of tiny blood vessels becoming clogged, causing portions of the retina to be in less oxygen, a condition known as retinal ischemia [49]. These retinal areas convey electrical signals to the body to stimulate the creation of new blood vessels and maintain oxygen. These aberrant blood vessels are unstable and quickly break, resulting in rapid vision loss from hemorrhage into the vitreous [50].

### Conventional approaches for treatment of diabetic retinopathy

Studies of anti-VEG for Diabetes mellitus have shown that the ranibizumab’s monthly use for thirty-six months has avoided the worsening of diabetic retinopathy and severity in the early stages of DR [51]. Various studies have also shown that corticosteroids could diminish the risk of the development of diabetic retinopathy, which includes the expansion of proliferative diabetic retinopathy. Many corticosteroids decrease the production of VEGF, decrease the breakdown of the barrier of the blood-retina, as well as perhaps have anti-angiogenic characteristics [52]. Furthermore, intravitreal triamcinolone acetonide has been utilized in inhibition of retinal neovascularization [53]. Nevertheless, just using corticosteroids to minimize the development of diabetic retinopathy has not been suitable owing to likely complications for example cataract and glaucoma as well as need for the reinjection because of the shorter duration of action. Because corticosteroids promote the progression of cataracts in phakic eyes, the visual outcomes may be compromised by the cataract formation. There have been a limited number of comparative studies comparing bevacizumab and triamcinolone acetonide. These have been predominantly small retrospective investigations with small follow-up periods [54, 55]. Kriechbaum et al. [56] compared 8 mg of triamcinolone acetonide to 2.5 mg of bevacizumab in a prospective comparative study. Visual improvements and macular thickness reduction were comparable between the two groups at six months. Bevacizumab demonstrated superior visual gains during the 12 months while the triamcinolone acetonide-treated eyes exhibited the formation of cataracts. Garbe et al. [57]. demonstrated that open-angle glaucoma and composite ocular hypertension were more likely to develop after long-term use of high-dose corticosteroids. Their discovery has prompted considerable concern among physicians, as glaucoma is a chronic eye disease that, if not properly treated, can result in permanent vision loss [58, 59].

In high-risk proliferative DR, a comparative study was performed to assess the effectiveness of intravitreal bevacizumab in conjunction with pan-retinal photocoagulation versus pan-retinal photocoagulation alone [60]. All neovascular recurrences occurred after the 4-week visit in the PRP-only group, whereas no recurrences occurred in the PRP-plus group; therefore, the authors recommend intravitreal bevacizumab be administered earlier in eyes with complete neovascular regression after PRP. An earlier PRP may provide some protection against neovascular recurrence following bevacizumab. An alternative investigation assessed the effectiveness of intravitreous aflibercept (anti–VEGF) injections in preventing vision-threatening complications in eyes with moderate to severe NPDR in comparison to sham treatment [61]. In this randomized clinical trial, periodic aflibercept decreased the proportion of eyes with moderate to severe NPDR that developed PDR or vision-reducing center-involved diabetic macular edema CI-DME in comparison to sham treatment.

Pan-retinal photocoagulation (PRP) is a therapeutic approach employed to control both proliferative and non-proliferative forms of DR. The Early Treatment Diabetic Retinopathy Study (ETDRS) and the Diabetic Retinopathy Study (DRS) are two randomized controlled trials that provide substantial evidence for the application of PRP [62]. The findings of DRS demonstrated that PRP decreased the incidence of severe vision loss in high-risk PDR, whereas ETDRS reported that PRP significantly decreased the incidence of high-risk PDR by 50% and laser application decreased the prevalence of moderate vision loss by 50% in patients with clinically significant macular edema.

Laser photocoagulation has been extensively employed for over 55 years as a treatment of several ocular diseases. Retinal laser therapy commonly employs either a 514 nm continuous wave (CW) argon or frequency-doubled 532 nm neodymium-doped yttrium aluminum garnet (Nd: YAG) solid-state lasers with 50 milliseconds pulse durations. However, Safety remains a primary concern with regard to such laser treatments [63]. Laser therapy, either as a focused or grid application for macular edema or as a PRP application for proliferative disease, may give a more persistent response, according to the results of certain clinical trials [64, 65]. Nevertheless, the conventional approach of using CW or photocoagulative laser causes damage to some retinal cells and frequently leads to the development of permanent scotomas within the visual field [66, 67]. Light-to-dark adaptation may be disrupted and night vision may be impaired by PRP [68–70]. The micropulse 810-nm diode laser was then utilized in a process known as subthreshold diode micropulse (SDM) photocoagulation. SDM is an effective treatment for proliferative diabetic retinopathy and DME, devoid of any adverse effects or complications, in small clinical trials [71, 72]. Laser treatment by using a diode laser is developed for minimizing the inevitable loss of visual field from the collateral injury of laser therapy using conventional continuous. RPE-targeting lasers such as SDM, selective retina therapy (SRT), and retinal rejuvenation therapy (2RT) could cause fewer injury to neural retina in DME as compared to the CW laser therapy, via utilizing a short pulse duration such as a nanosecond or a microsecond.

Numerous treatments in the starting stages of DR have been assessed. However, it should be suggested that more prudent and less aggressive treatments should be utilized to treat the early stages of DR to avoid side effects. Novel technologies in both functional assessments and retinal imaging, for example, OCT or UWFA, would allow the detection of early changes as well as for designing a personalized and noninvasive treatment. These efforts would be in effect in decreasing burden as well as refining the clinical outcome for the patient. Among the components of systemic treatment should be the regulation of serum lipids, blood sugar, and blood pressure. a) The incidence of DR is directly and consistently correlated with HbA1c (glycated hemoglobin) levels in glycemic control. It has been shown that effective glycemic control can decrease the occurrence and advancement of DR. Having the target of glycemic control HbA1C at 6% would be highly desirable. (b) An additional significant risk factor for developing and/or progression of DR is hypertension. Endothelial stress attributable to hypertension results in the secretion of VEGF, which disrupts the autoregulation of the retina and causes an increase in perfusion pressure and damage [73–76]. This risk factor is fortunately treatable. It would be preferable to have the target of hypertension treatment be 130/80 mmHg or lower (c) It is the role of the renin-angiotensin system to regulate blood pressure, as well as to participate in angiogenesis and retinal dysfunction. Localized production of angiotensin-converting enzyme (ACE) has been demonstrated by endothelial cells of retinal blood vessels and pigment epithelial cells of the retina [77]. Aqueous humor from patients with proliferative DR contains a high concentration of ACE [78]. It was discovered that the administration of ACE inhibitors, including lisinopril and candesartan, positively influenced the progression of DR [79, 80], which may be a viable option for diabetic patients with hypertension. (d) Dyslipidemia is positively associated with the development of diabetic retinopathy and macular edema. Dyslipidemia causes the formation of hard exudates [81]. Multiple studies have demonstrated the favorable effect of lipid-lowering drugs, such as atorvastatin and simvastatin, in decreasing hard exudates and retinopathy development [82, 83].

Ocular diseases have traditionally been treated through the administration of two main modalities: intravitreal injections and topical drops. However, adequately delivering therapeutic agents to the back of the eye (i.e., the retina) remains challenging. Consequently, new treatment options have emerged to alleviate these side effects of existing conventional therapies and reduce the therapeutic burden of monthly intravitreal injections. These developments have provided hope to patients with advanced DR, as it is estimated that 95% of these patients could maintain their vision if treated before severe retinal damage [84]. These include novel drug delivery systems based on nanoparticles, sustained delivery of therapeutic agents, and targeted delivery of drugs to specific cells or tissues; they also include enhanced delivery of water-insoluble and large biomolecule pharmaceuticals [85]. Several nanomaterials have the potential to enhance the safety and efficacy of existing treatments, therapies, and surgical procedures associated with retinal diseases, as well as to accelerate and improve the accuracy of retinal disease diagnosis. The efficacy and safety profiles of drug delivery systems utilizing nanomaterials have been superior to those of other drug delivery systems in vitro and in vivo models. Nanomaterial-based delivery systems have exhibited superior bioavailability in comparison to alternative means of administration, as well as prolonged release and decreased dosing or injection frequency [86–88]. Many innovative approaches based on neurons, vascular pathology, and retinal glial during diabetes are being developed, however none have yet reached clinical usage.

## Nanocarriers for treating diabetic retinopathy

**Fig. 2:**
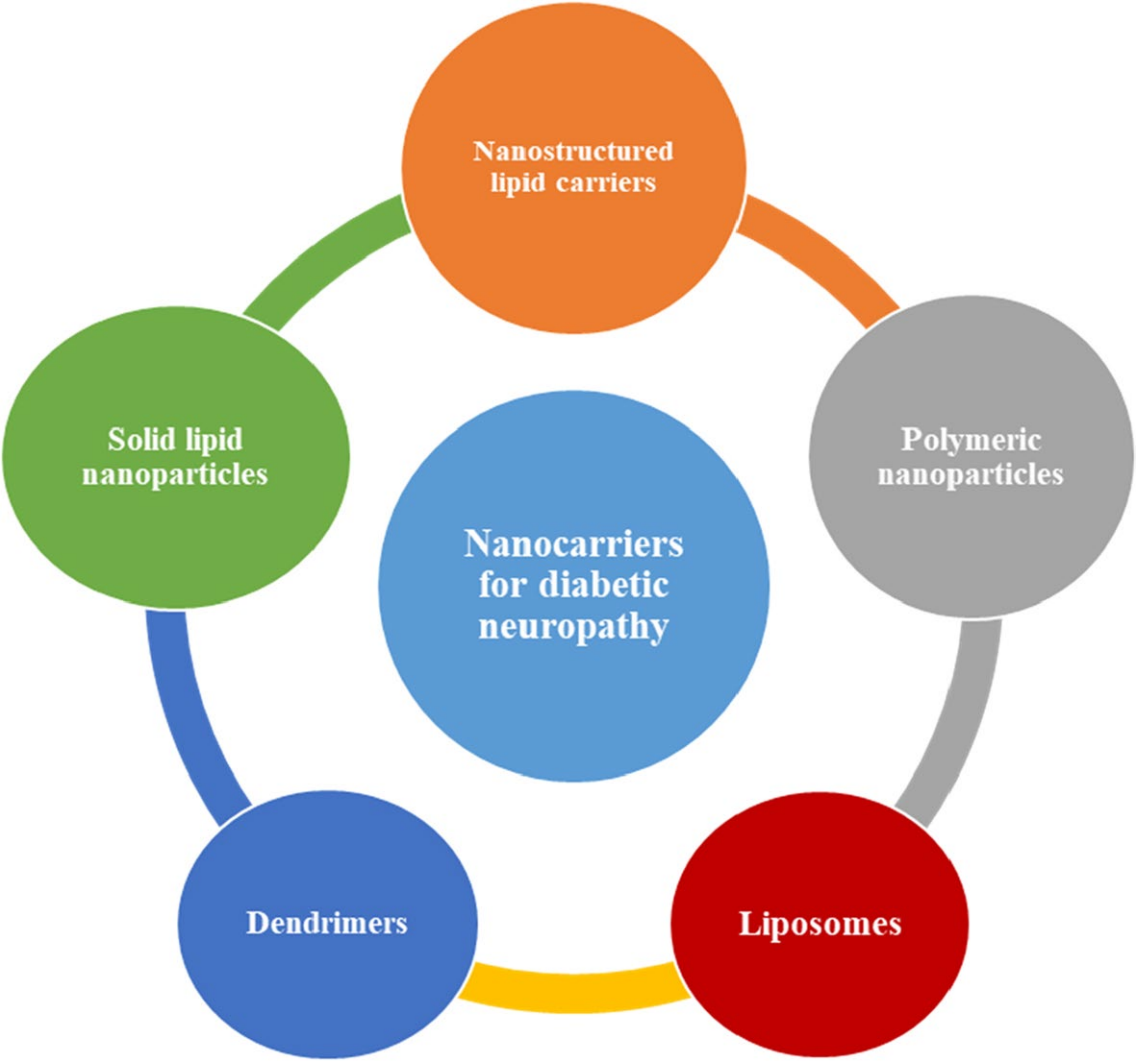
Schematic illustration of nanocarrier utilized for the treatment of DR

Nanoparticles may reduce injection frequency while improving efficacy, resulting in better therapeutic effect [89]. Furthermore, nanoparticles can be constructed of muco-adhesive polymers, increasing the time of ocular permeation and allowing them to stick to the sclera’s surface while resisting washing by tear fluid [90, 91]. The nanoparticles also function as drug stores, allowing the medications to be released continuously throughout time [92]. Using nanoparticles for gene therapy or controlled-release medicines can mitigate these adverse effects [93]. The use of nanoparticles in therapy allows for the targeting of retinal inflammation and other factors that contribute to the development of retinopathy [94]. Figure 2 shows various types of nanocarriers including nanostructured lipid carriers, solid lipid nanoparticles, dendrimers, liposomes, and polymeric nanoparticles that have been utilized in the treatment of diabetic retinopathy. Polymeric nanoparticles comprised of several polymers, such as poly(lactic acid) (PLA), chitosan, polyvinyl alcohol (PVA), poly(lactic-co-glycolic acid) (PLGA), and poly(methyl methacrylate) (PMMA) which have been approved by Food and Drug Administration (FDA) due to their biodegradability and biocompatibility [10]. Lipid nanoparticles, such as nanostructured lipid carriers (NLC), solid lipid nanoparticles (SLN), and liposomes, have been proposed for ocular delivery [95, 96]. Their composition consists primarily of physiological lipids, including phospholipids, ceramides, and glycerides, which are biocompatible and minimally or non-toxic [97, 98]. Dendrimers derive from polymers, usually comprised of water-soluble polyamidoamine (PAMAM) [99]. The importance of the described nanocarriers towards DR is illustrated in the following sections.

### Polymeric nanoparticles

These are made of a polymer solid matrix, and the medication is potentially adsorbed on the surface or disseminated within the particles’ core. These are made by polymerizing synthetic monomers or dispersing natural macromolecules or synthetic polymers. PLGA, chitosan, PLA, and polyvinyl alcohol, are the most often utilized polymers in the formulation of polymeric nanoparticles. The FDA has authorized these polymers. PLGA is commonly used for ophthalmic use [100] due to its non-immunogenicity, biocompatibility, non-toxicity, and biodegradability. Chitosan nanoparticles are particularly appealing in topically applied polymeric drug delivery systems for ocular applications [101]. Natural polymer, chitosan is relatively cheap, harmless, and biodegradable [102]. Because of the ionic contact that exists between the positive amino acids of chitosan and the ions that are charged negative residues of sialic acid of mucin thus possesses a high mucoadhesive characteristic [103]. As a result, a chitosan nanoparticle has a longer retention duration and better ocular membrane penetrability [104]. Polymeric nanoparticles provide regulated and prolonged medication distribution while also protecting the drug from acidic or enzymatic breakdown. Polymeric nanoparticles allow for the modification charge and size of particles. The limitations of polymeric nanoparticles include aggregation of particles, purification and handling issues, a restricted drug load capacity, and initial burst drug release. Attia Shafie et al., 2013 [105] prepare polymeric nanoparticles to provide medication in a controlled and prolonged manner while also shielding the substance from acidic or enzymatic degradation. Their drawbacks include aggregation of particles, purification, a limited drug load capacity, and early burst drug release. In general, nanoparticle-based systems employing natural polymers exhibited favorable biocompatibility and strong adhesive characteristics, facilitating substantial drug retention and permeation across ocular tissues while preventing the induction of toxicity. Nevertheless, natural polymers are susceptible to rapid degradation, and their production process is hindered by limited batch-to-batch repeatability. Synthetic polymers exhibit greater stability in comparison to natural polymers owing to their reduced biodegradability rates, which facilitate a prolonged and gradual release of pharmaceutical compounds. Conversely, synthetic polymers are deemed to be less mucoadhesive than their natural counterparts because they lack functional groups capable of interacting with the mucin layer [106]. To address the challenges posed by low mucoadhesiveness and rapid degradation, synthetic polymers have been incorporated with natural polymers such as chitosan, which possesses exceptional mucoadhesive properties [107]. Besides these, polymeric nanoparticles have certain other limitations, such as slow clearance and toxicity [108].

### Lipid-based nanoparticles

These were designed to get past the drawbacks associated with polymeric nanoparticles. As compared to polymeric systems, SLN consists of solid lipids, which means that the lipid matrix remains solid at both room as well as body temperature. The problem is the sensitive nature of lipids to the eye, which limits their applicability in ocular medication administration [109]. The European and US Food and Drug Administration have approved a wide range of lipids that are generally considered safe (GRAS) [96]. Among these lipids are triglycerides caprylic triglycerides, glyceryl palmitostearate, and monoglycerides (glyceryl monosterate) [110]. One of the primary advantages of SLN is that it is free of biotoxicity because it is made from physiological lipids, prolonged residence time, and improved ocular bioavailability [111].

In a research conducted by Fangueiro et al., 2014 [112] double emulsion approach was used to disperse cationic lipid nanoparticles for the entrapment of epigallocatechin. Lipid nanoparticles derived from water in oil-in-water emulsion are flexible colloidal carriers for peptide/protein and hydrophilic drug delivery [113]. The use of cationic nanoparticles increases the retention of drugs in the ocular mucosa while simultaneously targeting the posterior portion of the eye [242]. In vitro HETCAM testing revealed no eye discomfort. Ophthalmic irritation and pharmacokinetic characteristics were studied in vivo on rabbits. Due to their anti-inflammatory and anti-oxidant properties, lipid nanoparticles of epigallocatechin are effective for treating diabetic retinopathy [114].

In a study conducted by Li et al., 2013 [115] tetrandrine cationic SLNs were produced and tested for ocular medication delivery. Solid and cationic SLNs of tetrandrine were created using a low-temperature emulsion evaporation-solidification process [116, 117]. In vitro drug release tests revealed that they released the medication slowly. The cytotoxicity investigation revealed that solid lipid nanoparticles exhibited minimal cytotoxicity. As a result, tetrandine-loaded nanoparticles were a better strategy for ocular drug administration.

However, because of their rigid crystalline matrix, SLNs show some drawbacks. The main disadvantages of SLNs are the drug’s inevitable expulsion upon crystallization while storage and the moderate encapsulating capacity caused by the low solubility of drugs in the lipid [118, 119]. After storage and polymorphic changes in solid lipid structures, drug molecules in SLNs tend to be oriented between fatty acid chains or glycerides. Consequently, drugs that were previously dissolved in SLNs may experience expulsion during storage [120]. The crystalline structure of the solid lipid contributes to additional drawbacks, including unpredictability of gelation and intrinsically low incorporation rates [121].

### Nanostructured lipid carriers (NLC)

To solve the limitations of solid lipid nanoparticles, the next generation of lipid nanoparticles has been developed. NLC is made up of liquid and solid lipids, surfactants, and medicines. The addition of lipids in liquid form into the lipid solid matrix produces a structure with crystal defects, allowing for the addition of more medicines while maintaining the physical stability of the nanocarrier. In comparison to solid lipids, liquid lipids operate as a better drug solubilizer. Among the several nanocarriers, NLC has proven to be an effective ophthalmic medication delivery technology. Other advantages of using these are entrapment of non-hydrophobic drugs, which prolongs the encapsulated residence time of the drug and protection from degrading enzymes, and improved adhesion to the surface of the eye [122].

In a research conducted by Liu et al., 2012 [123] mangiferin nanostructured lipid carriers (NLCs) were designed to increase ocular bioavailability. Oxidative stress has been associated with ophthalmic diseases such as diabetic macular degeneration, cataracts, and macular degeneration related to age [124] and mangiferin is a powerful antioxidant [125]. On the ocular surface and in the conjunctiva, the improved formulation demonstrated a longer residence time. Finally, a pharmacokinetic investigation of mangiferin nanostructured lipid carriers versus mangiferin solution revealed that mangiferin NLC had higher bioavailability (5.59 fold) than mangiferin solution. As a result, the NLC system could be used to improve mangiferin bioavailability. Araujo et al., 2010 [126] Triamcinolone acetonide-loaded nanostructured lipid carrier was created for ocular antiangiogenic applications [127]. The Draize eye test revealed that the optimized formulation of NLC is not hazardous or irritating to the ocular tissues and proves that an effective medication delivery strategy for retinal illnesses is a possibility.

NLCs, despite their considerable potential, encounter specific constraints such as cytotoxic effects associated with the composition and concentration of the matrix, irritant and sensitizing properties of certain surfactants, and challenges in the application and efficiency of protein and peptide drugs as well as gene delivery systems that require further investigation [128].

### Liposomes

These are small spherical vesicles of lipids composed of cholesterol and phospholipids. Phospholipids are biocompatible, non-toxic, and biodegradable, which has sparked interest in them as an ocular drug delivery carrier [129]. Abrishami et al., 2009 [130] created bevacizumab liposomes. The rehydration dehydration process was used to create bevacizumab liposomes. Morphological examinations revealed multi-lamellar vesicles. The animal models can be utilized to investigate bevacizumab and bevacizumab liposomes’ vitreous and aqueous concentration-time profiles. After 42 days of testing, liposomes of bevacizumab demonstrated higher concentrations in vitreous than non-liposome formulation. In a study by Kaiser et al., 2013 [131] Minocycline nanoliposome formulation was created for treating DR. In the rat model, systemic treatment of minocycline was demonstrated to be effective in DR by lowering retinal pro-inflammatory cytokine production and activation of microglia. However, as with most antibiotics, long-term systemic use carries severe dangers. As a result, the minocycline nanoliposome formulation delivered by subconjunctival injection was appropriate for delivering medicines into the back portion of the eye to treat DR.

Although liposomes offer various benefits, they also have certain drawbacks such as instability and an increase in particle size during storage, vulnerability to being absorbed by phagocytes, and a limited duration of retention in the retina [132, 133]. Liposomal phospholipids are susceptible to hydrolysis and oxidation reactions, both of which can lead to undesirable consequences. The body utilizes various defense mechanisms (reticuloendothelial System, opsonization, and immunogenicity) to detect, counteract, and ultimately eradicate liposomes, similar to its response to any extracellular particle that penetrates the body. Although it is essential to overcome these challenges to achieve optimal liposome performance, it is possible to take advantage of other factors, such as the enhanced permeability and retention (EPR) effect, to improve drug delivery [134].

### Dendrimers

These are a new family of synthetic nanoparticles made from polymers having a structure tree that is extremely branched in three dimensions. Dendrimers are nanometer-sized particles with diameters ranging from 2 to 10 nm. Dendrimers are distinguished by their star size, form, monodispersity, and molecular weight. Various series of poly (amidoamine) dendrimers were tested for regulated ocular medication delivery by the researchers [135]. Dendrimers possess numerous benefits, including the ability to encapsulate poorly water-soluble drugs within their internal cavities, maintain a low polydispersity index and dendrimer size, prevent uptake by the reticuloendothelial system (RES), enhance solubility, permeability, and retention effect, and dendrimers with anionic and neutral surfaces lack cytotoxicity and ocular irritation while maintaining targeting efficiency [136]. Several studies claimed that dendrimers were employed in ophthalmology for a variety of reasons including medication delivery, delivery of genes, peptides delivery, biological, genetic testing, and imaging.

Kambhampati et al., 2015 [137] created dendrimer-related compositions aimed at retinal microglia. The retinal absorption dendrimer coupled with cyanine dye (D-Cy5) was examined in normal and I/R mice eyes. Immunofluorescence was used to assess uptake of the dendrimer using the Iba-1 antibody of rabbit with a Cy3-tagged secondary antibody (microglia/macrophage). Fluorescence spectroscopy was used to measure absorption in the retina and other organs. Dendrimers target active microglia and have essentially identical retinal distribution when supplied via either route.

Yavuz et al., 2015 [138] PAMAM dendrimer complexes with dexamethasone (PAMAM) were tested for DR. Dexamethasone dendrimers were produced and characterized using several PAMAM polymers. The particle size spans between 49 and 425 nm. Permeability, cytotoxicity, ex vivo transport, and ocular pharmacokinetic studies were performed on the formulations. Cell viability was observed to be 87.5% with dexamethasone and 73.5% with the combination. Different polymers have been utilized for the manufacturing of nanocarriers for treating DR and have been described in Table 1, along with their therapeutic outcomes.

Despite these benefits, dendrimers have some drawbacks, including, depending on the materials used and the method of production, high expenses for synthesis, elimination, and metabolism. Moreover, their cellular toxicity has not been sufficiently evaluated [139, 140]. Furthermore, the increased toxicity of carbosilane dendrimers of higher generations (G4–G6) typically restricts their application as conjugates with pharmaceuticals [141]. Dendrimer synthesis is a simple process in theory. But in practice, it’s a laborious and time-consuming process [142, 143]. Furthermore, despite employing extremely selective reactions, the ultimate yield achieved during dendrimer production is often inadequate. The synthesis process receives significant expenses and the acquisition of high-generation dendrimers devoid of structural defects presents additional drawbacks.

**Table 1:** Various studies of nanocarriers of various polymers for the treatment of diabetic retinopathy

Type of nano carrier	Lipid or polymer used	Drug	Method	Particle size range (nm)	Evaluation	Results	References
Polymer	Chitosan sodium alginate	Betamethasone sodium phosphate	Ionotropic gelation	168–692	In-vivo and *in-vitro* studies	Better *in-vivo* and *in-vitro* skin permeation studies.	[144]
Polymer	Chitosan	Bevacizumab	Emulsion evaporation	88.9 ± 106.7	*In-vivo* and *in-vitro* studies	These inhibit VEGF expression in rat retina and show a long duration of action	[145]
Liposo mes	Cholesterol	Bevacizumab	Dehydration rehydration	80 − 20	*In-vivo* cytotoxicity and transport	When compared to non-liposomal formulations, these achieve higher concentrations in the retina.	[130]
SLN	Cetyltrimethyl ammonium bromide	Epigallocat echin gallate	Multiple emulsion	Less than 300	*In-vivo* and *in-vitro* release studies	There was no eye discomfort and first-order kinetic release with epigallocatechin-loaded cationic lipid nanoparticles.	[98]
NLC	Precirol ATO 5 squalene	Triamcinolone acetonide	Homogenization	100 to 300	*In-vivo* and *in-vitro*	The study with Triamcinol one loaded NLC found no eye harm or discomfort.	[146]
Polymer s	Chitosan	Curcumin	Coacervation	61.5 to 90	*In-vivo* and *in-vitro* studies	These were found to be nontoxic, nonirritant, and to have a better mucoadhesive action. Curcumin-loaded nanoparticles inhibited cell proliferation in the rabbit eye.	[147]

## Chitosan as an advanced material for the drug delivery system

**Fig. 3:**
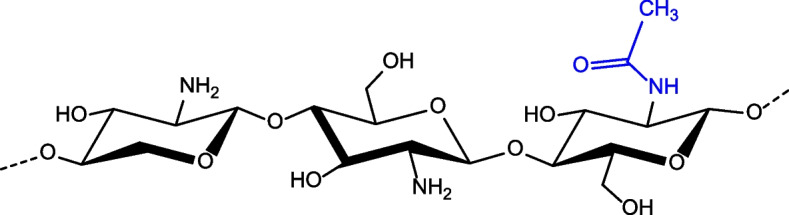
Structure of Chitosan

Chitosan (CS) is a commonly used polymer and it is also 2nd most common polymer after cellulose, a naturally existing amino polysaccharide [148]. It is synthesized in an alkaline environment from chitin (found in the exoskeletons of insects and marine aquatic creatures, as well as microorganisms like fungi, yeast, and microalgae) [149]. It is a linear polysaccharide made up of randomly distributed − (14)-linked D-glucosamine (acetylated unit) and N-acetyl D-glucosamine (deacetylated unit) (Fig. 3).

The molecular weight (MW) of CS ranges from 300 to 1000 kD according to the source and preparation processes, with a deacetylation degree of 30–90%, DD [150]. Chitosan possesses remarkable chemical and biological properties, which have led to its widespread use in pharmaceutical and biomedical applications such as drug delivery, delivery of genes, tissue engineering, and so on. Because of its polycationic nature, CS is soluble in water and a bioadhesive that rapidly adheres to surfaces with negative charges like mucosal membranes [151].

**Fig. 4:**
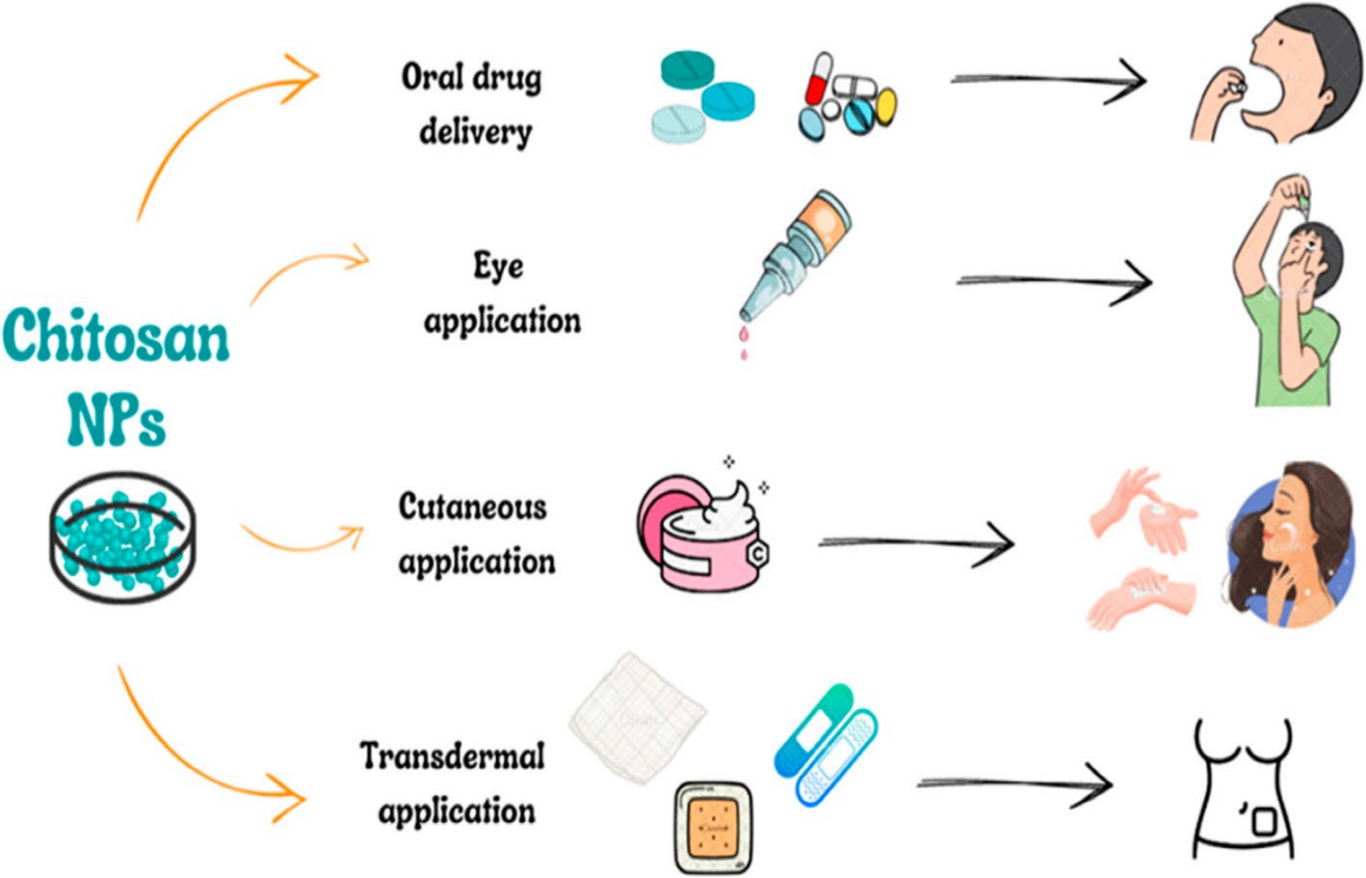
Various drug delivery applications of Chitosan nanoparticles

CS has solubility in aqueous and acidic solutions at ambient temperature, no harmful organic solvents or heat are required for the synthesis of CS NPs. Small compounds, proteins, and polynucleotides are just a few of the medications that can be included in CS drug delivery systems (DDS) [152]. The controlled release of the encapsulated medication is possible with CS [153]. This is advantageous for medications that have inadequate plasma levels and oral delivery and must be administered parentally [154]. It is also used for transporting medications that are vulnerable to metabolic breakdown in the GI system, resulting in increased efficacy and patient compliance [155]. Multiple applications of chitosan nanoparticles have been shown in Fig. 4.

Because of a few fundamental beneficial characteristics, for example, bioactivity biocompatibility and to some extent, target specificity, polymeric nanoparticles made from chitosan (CS NPs) are acting as an excellent drug carrier [156]. These have a high mucoadhesive capability, increased bioavailability, and a high rate of dissolution for non-lipophilic medicines [157]. With oral medication delivery, their limited solubility at physiological pH impacts intake. In general, the loading effectiveness of CS alone to encapsulate water-insoluble medicines is rather poor [158, 159]. Multiple advantages and disadvantages of chitosan nanoparticles are mentioned in Table 2.

**Table 2:** Advantages and disadvantages of chitosan nanoparticles

Advantages of nanoparticles of chitosan	Disadvantages of nanoparticles of chitosan
Less toxic	Possible contraction
Improve biocompatibility	Less mechanical resistance
Muco-adhesiveness	Low solubility in alkaline and neutral pH
Targeted drug delivery	Difficult pore size control
Stability	Effect of cross-linking on chitosan
Increase therapeutic efficacy	Problem in electrospinning

CS NCs are Vesicular structures in which the medication is contained in a cavity made up of an oily core surrounded by a CS shell [160]. Castro et al. [161] investigated the biological and physicochemical characteristics of docetaxel (DCX) packed nanocapsules of chitosan modified with the chimeric mAbs ( monoclonal antibody) as a potential cancer therapy improvement treatment. The NCs, which were created as an oil core surrounded by a polymeric shell, enabled a 99.9% encapsulating effect of DCX.

## Chitosan-based nanocarriers for the treatment of diabetic retinopathy

**Fig. 5:**
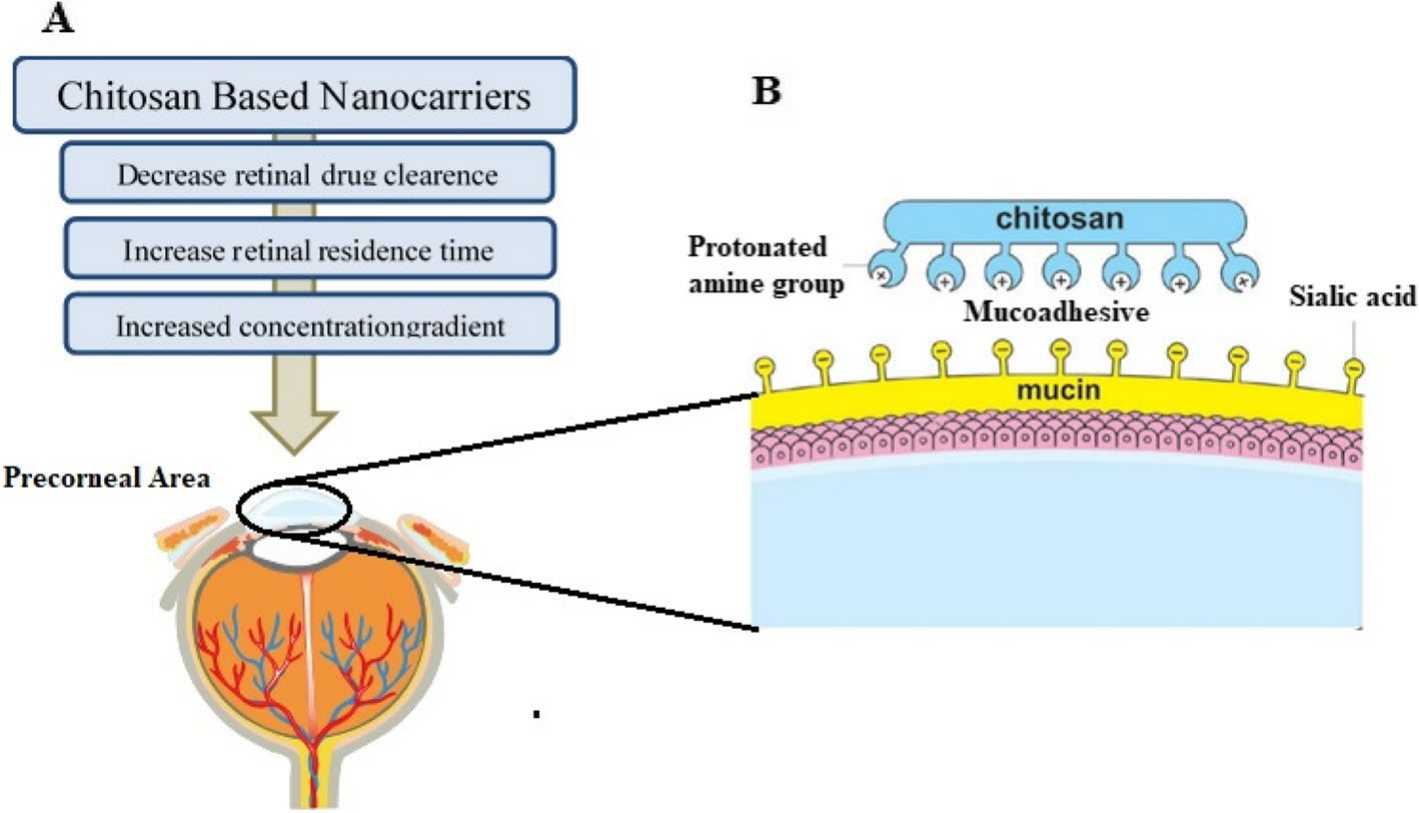
Advantages of CS-based nanocarriers for the treatment of DR (**A**) and mucoadhesive character of chitosan, illustrating the interaction between protonated amine groups of chitosan with the negatively-charged sialic acid residues present on the mucin layer of the cornea. Reproduced and modified from [172] under the terms of the Creative Commons Attribution License (https://creativecommons.org/licenses/by/4.0/)

Treatment for diabetic retinopathy can be achieved by enhancing the transport of drugs to the retina following topical application. Topical administration corresponds to the process of applying medication onto the tear film surface of the eye. This particular route is extensively employed for the delivery of drugs to address eye diseases affecting the anterior segment. However, topical drug delivery for the treatment of diseases affecting the posterior segment, such as AMD and DR, continues to be a significant challenge [162]. Hence, various research has been dedicated to utilizing this method of drug administration to deliver drugs to the posterior segment of the eye. Prolonged contact between drug delivery systems and the ocular surface, sustained drug release, and improved corneal permeability are the primary objectives of ocular drug delivery. Due to the aforementioned factors, a wide range of approaches have been developed to administer ocular drugs to the posterior segments of the eye, including the use of mucoadhesive nanoparticles for ocular drug delivery [163, 164]. Conventional eye therapy’s key drawbacks are limited residence time, drug drainage, and repeated instillation [165]. Mucoadhesive nanostructures have been designed to overcome the problems related to conventional therapy. The capacity of mucoadhesive polymers to attach to and interact with the mucosal layer of the tear film is utilized in mucoadhesion for ocular administration. An estimated 5–10% of the total drug quantity that surpasses precorneal clearance may penetrate the eye, with the precise percentage dependent upon the permeability coefficient and molecular weight of the drug [166]. Mucoadhesion is presently being investigated for its potential to enhance the probability of corneal and conjunctival absorption to the posterior segment after topical administration, thereby facilitating non-invasive drug delivery to the posterior eye segment, due to its capacity to inhibit rapid precorneal clearance and prolong precorneal residence time. CS has been the subject of extensive investigation among the various types of polymers in regard to enhancing topical drug delivery specifically to the posterior eye segment [167]. CS is commonly characterized as a highly mucoadhesive polysaccharide that can form a strong connection with the mucus layer covering the eye mucosa via electrostatic interaction between protonated amine groups of CS and the negatively-charged sialic acid residues present on the mucin layer Fig. 5. CS nanoparticles easily enter conjunctival epithelial cells and are well tolerated at the rabbit ocular surface [168]. These carriers have the potential to be used as a medication delivery method for the ocular mucosa. Recently, hyaluronic acid and chitosan nanoparticles with low cell toxicity and the ability to infiltrate epithelial cells of the cornea via CD44 receptor endocyte uptake have been discovered [169]. These Nano systems have been designed specifically for the transport of hydrophilic and lipophilic medicines, as well as polynucleotides, to the eye’s surface [169]. Chitosan and alginate polymers were chosen for this investigation because of their mucoadhesiveness and non-toxicity [170]. The physicochemical characteristics and in vitro release of betamethasone-loaded polymeric nanoparticles were investigated [171]. The first burst release was followed by persistent release in vitro. Rabbit sclera was used in in vitro permeation investigations. On the rabbit model, in vivo permeability studies were performed for the improved formulation. According to the findings of this study, nanoparticles of alginate and chitosan with betamethasone drug delivery would be successful in targeting the posterior portion of the eye [171].

**Fig. 6:**
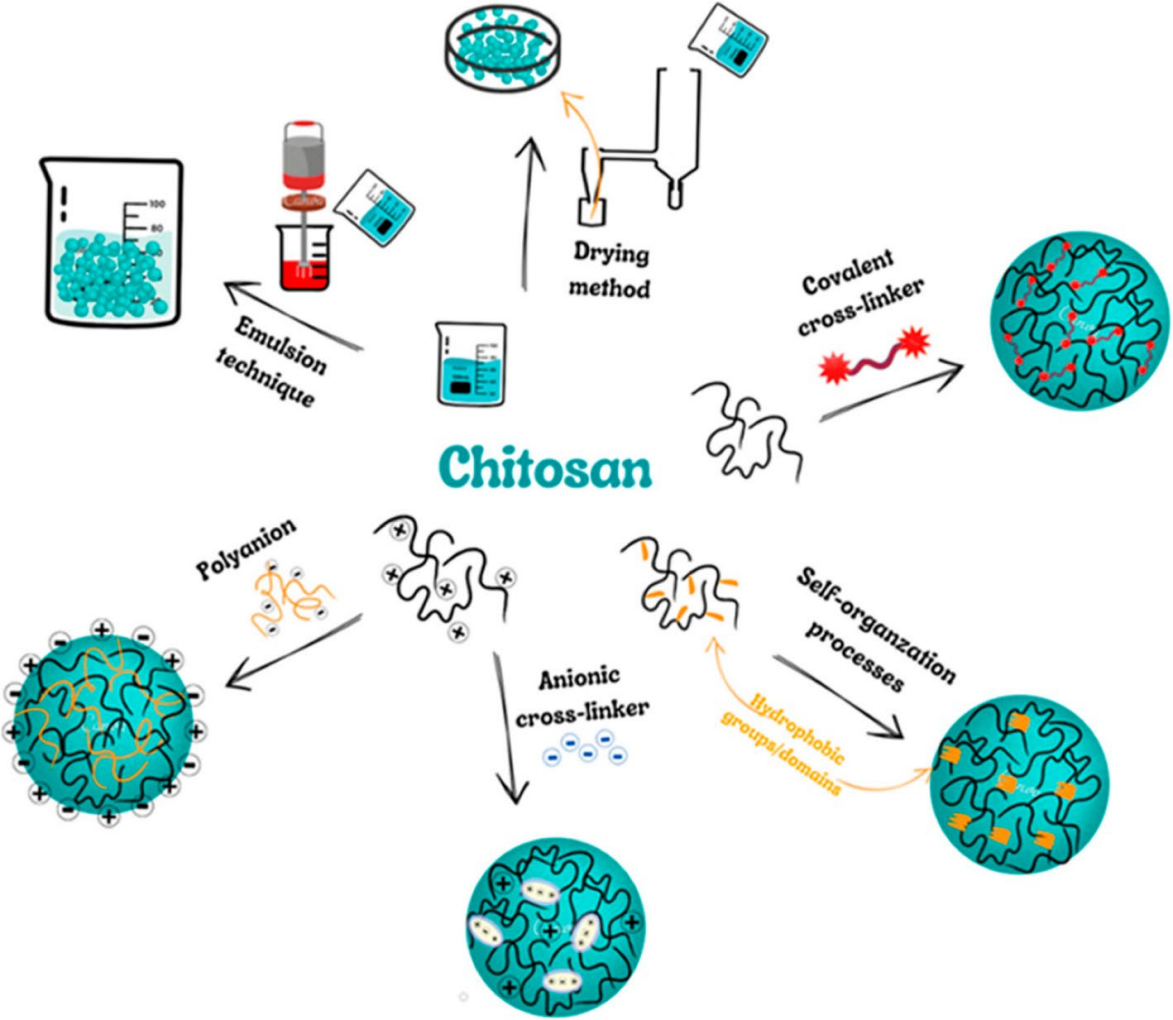
Methods for manufacturing of chitosan nanoparticles. Reproduced from [175] under the terms and conditions of the Creative Commons Attribution (CC BY) license (https://creativecommons.org/licenses/by/4.0/)

Deepa Pathak et al., 2014 [173] developed a polymeric nanoparticulate system for ocular administration employing plant-based anionic polymer. Up to 2000 µg/ml, the prepared nanoparticles exhibited no irritant or adverse effects in vitro and in vivo. Yan Lu et al., 2014, examined the bevacizumab chitosan nanoparticles [174] in the therapy of DR. Nanoparticles of chitosan were designed and studied to address the issues related to anti-VEGF therapy. Photo correlation spectroscopy was used to determine the particle diameter of bevacizumab-chitosan nanoparticles, which was 88.9 ± 106.7 nm. The findings demonstrated that bevacizumab inhibited VEGF expression effectively, and the duration of action of bevacizumab-chitosan nanoparticles was longer than that of bevacizumab alone. Various methods for the manufacturing of chitosan nanoparticles in various studies have been shown in Fig. 6.

Rong X et al. created and reported effective results for a novel insulin delivery method by making hydrogel of CS nanoparticles/ poly lactic co glycolic acid- poly ethylene glycol-poly lactic co glycolic acid (ICNPH) [176]. There was no apparent impairment observed in the structure, function, or neurons of the retina. ICNPH revealed sufficient neuroprotective impact on retinas in DR rats via subconjunctival injection and allows regulated insulin delivery. Badiee et al. developed a method that included bevacizumab chitosan nanoparticles inside a hyaluronic acid ocular implant. The results showed that this one-of-a-kind composition was capable of sustained medication release for two months. As a result, the use of this formulation represents a promising technique for obtaining prolonged bevacizumab administration [177].

Mukhopadhyay et al. [178] reported the synthesis of chitosan and alginate nanoparticles loaded with insulin by mixing insulin and CaCl_2_ with 0.1 M HCl to the produced alginate solution, creating ionic polyelectrolytes following adequate sonication. Following this, chitosan polyelectrolyte complexation was performed by adding chitosan solution with gentle stirring to form core-shelled nanoparticles. The produced nanoparticles had 100–200 nm particle size and were able to provide pH-responsive sustained release and encapsulation effectiveness of 85%. Hepatotoxicity tests such as ASAT and ALT revealed that neither the liver had been impaired nor the functioning of the liver was impaired.

Bhattacharyya et al. [179] synthesized core-shell tiny carriers for oral insulin administration. Instead of utilizing pure alginate, they used a homogeneous mix of polymer called alginate (PU-Alg) to create the core of the required nanoparticles. The PU-Alg core and chitosan shell nanoparticles were then prepared by adding CS solution and sonicating for another 15 min. The produced nanoparticles exhibited a small particle size of 90–100 nm, a high encapsulation efficiency, extended reduction in glucose level (up to 98 mg/dL for a 100 IU/kg insulin injection at the 10th hour), and enhanced insulin bioavailability.

Chen et al. used a polyelectrolyte combination of CS and alginate to build a modified system as a second step [180] after manufacturing nanoparticles loaded with insulin using the double emulsion water in oil in water type. Insulin-loaded w/o/w nanoemulsions of coated chitosan and alginate were produced. The produced chitosan -coated and alginate -coated nanoparticles had encapsulation efficiencies of 81.5 7.4% and 55.2 7.0%, respectively, with an average particle size range of 200–300 nm [181]. It may be concluded that adding a polyelectrolyte complex step increased the level of glucose reduction by three fold for a period of up to 12 h by altering insulin release throughout the GIT.

Mahaling et al. conducted a study in a DR-induced rat model using triamcinolone acetonide-loaded nanoparticles [182]. After 20 days, there was a significantly decreased inflammation of the retina, as demonstrated by the decreased expression of NF-B, ICAM-1, and TNF. It significantly reduced microvascular problems, as seen by lower VEGF production and fewer microvascular tuft forms after 40 days of treatment. Investigations on albino male rats revealed dexamethasone levels comparable to those recorded in the FDA-approved NP system with dexamethasone combined with castor oil. Preocular injections such as intravitreal injections, extend the duration of drug delivery, but with the additional advantage of a site of injection that reduces the likelihood of particle leakage due to rupture.

Existing DR treatment options are costly, intrusive, and require specialist administration. As a result, a topical preparation that can permeate deep into the back section of the retina is required. Furthermore, it should provide long-term relief. In recent years, innovative drug delivery systems have been investigated and proved to be effective in the treatment of DR. Nanostructured lipid carriers of chitosan-modified 5- fluorouracil (CS-5-FU-NLCs) were synthesized using a Box-Behnken Design. Ex vivo permeation studies and in vitro drug release indicated that these have higher and sustained drug release than the 5-FU solution. Animal models ensured that they were non-irritant. In vivo ocular tests using CS-5-FU-NLCs verified the antiangiogenic efficacy of 5-FU in a CAM model and a DR-produced model of rats, demonstrating that 5-FU was successfully delivered to the retina.

Zeng et al. [183] created PLGA and chitosan nanoparticles incorporating interleukin-12, a cytokine for suppressing tumor angiogenesis and lowering levels of VFGF-A and MMP-9. Despite the formulation’s low entrapment efficiency (34.7%), it shows a higher therapeutic effect in suppressing VEGF-A and MMP-9 production in endothelial cells of rats and retina of diabetic retinopathy-induced mice. The improved thickness of the retina and reduced neovascularization after therapy demonstrated that this formulation exhibited negligible damage to the retina in DR animals.

A novel insulin delivery system was developed which contains chitosan nanoparticle hydrogel to pursue insulin sustained release system to cure DR. Real-time polymerase chain reaction, HE staining, and transmission electron microscopy characterization tests were used. Subconjunctival injection was administered which reduced the DR in rats. The findings showed that ICNPH has a neuroprotective impact in DR rats by subconjunctival injection and promotes regulated insulin delivery. In the near future, it could be one of the therapy options for DR [184].

In general, nanoparticles-based CS demonstrates effective adhesion to the ocular surface following application and can penetrate the corneal and conjunctival epithelium. CS-based nanoparticles exhibit limited penetration but exhibit an extremely strong interaction with the ocular mucosa, ultimately resulting in enhanced delivery and transportation of the encapsulated molecules. Furthermore, it has been noted that the conjunctiva is the preferred site for CS nanoparticles. Moreover, Lipid CS-nanoparticles exhibit a more pronounced affinity for the conjunctiva. In this particular instance, the overall performance of the nanosystems in relation to their interaction with the mucus layer that envelopes the conjunctival epithelium is influenced by the lipidic homologue. Overall, the interaction of nanostructures based on CS with the superficial tissues of the eye, including the cornea and conjunctiva, seems to be influenced by their specific affinity for both mucus and epithelial cells. Nevertheless, further mechanistic investigations are crucial to acquire a thorough understanding of the challenges associated with this interaction [185].

## Challenges and future perspectives

Intravitreal anti-VEGF pharmaceuticals and surgical intervention are the prevailing current therapies for posterior segment ocular disorders, including diabetic retinopathy and DME [186, 187]. Ocular bioavailability is inadequate in conventional dosage formulations [188]. Consequently, intraocular invasive procedures, such as intravitreal injections, are necessary to deliver large quantities of any substance to the posterior segment of the eye but these methods necessitate recurrent and consistent administrations for several months to a year [186]. Therefore, studies focused on developing better methods of ocular drug delivery that allow for less intrusive routes and fewer dosages, accurate drug targeting within the eye, increased therapeutic efficacy, decreased side effects, and improved patient compliance became a top priority [189]. In this respect, there have been promising investigations on CS-based mucosal delivery methods for enhancing topical drug delivery to the posterior segment of the eye. In conclusion, the elements and challenges must be taken into account to effectively implement Cs-based mucoadhesive DDSs for posterior ocular drug delivery into clinical practice.

Designing a mucoadhesive drug delivery system requires a comprehensive evaluation of multiple parameters. An important aspect of consideration is the degree of mucoadhesiveness of the formulated drug delivery system. Developing a system with exceptional mucoadhesive properties and the ability to resist rapid clearance is an exceedingly desirable goal. Significant determinants of a polymer’s suitability for mucoadhesion include molecular weight, chemical structure, concentration, surface charge, surface tension, and rate of hydration. Regarding ocular mucoadhesion, it is important to thoroughly demonstrate safety, absence of irritation, no adverse effects on visual activity, and a high capacity for loading drugs. Additionally, it is crucial to ensure complete drug release and assimilation before the turnover of ocular mucin, which occurs every 15–20 h [190]. CS was utilized in the majority of the reviewed literature that examined mucoadhesion for posterior eye delivery due to its safety, abundance, cationic properties, biocompatibility, biodegradability, and lack of immunogenicity [191]. The maintenance of chitosan in its soluble and positively charged state, which is essential for preserving its mucoadhesive and permeation enhancement properties, has been observed in an acidic environment [192]. Therefore, it is critical to recognize that when the chitosan-based drug delivery system is applied to the neutral pH of the ocular surface, the mucoadhesive and permeation enhancement properties of chitosan are anticipated to be significantly compromised. In certain comparable circumstances, slightly acidifying the formulation could serve as a potential remedy. These formulations can lead to increased lachrymation and reflex blinking as a result of the hypersensitivity of the ocular tissues, this will ultimately enhance precorneal clearance and the elimination of the dosage form from the ocular surface. Moreover, the ability of human tears to buffer may challenge this premise. Another crucial factor to address when translating CS-based systems into clinical use is the verification of their safety and biodegradability. Due to its cationic nature, CS is anticipated to have adverse impacts on both the corneal and conjunctival tissues. Therefore, a considerable number of research studies conducted both in vitro and in vivo have examined the toxicological characteristics of chitosan-based systems for delivering drugs to the eyes [168, 193, 194]. It is crucial to indicate that the safety profile of CS-based systems is dependent on both concentration and composition. For example, numerous studies have documented CS-based systems that exhibit nearly 100% cell viability and survival, devoid of any indications of in vivo inflammation or modification [168, 194, 195]. Nevertheless, the modest level of toxicity seen was directly associated with the low amounts of CS, which did not surpass 2 mg/mL. Another crucial factor to consider is the impact of particle composition on toxicity. CS liposome complexes exhibited greater cell viability compared to chitosan nanoparticles following a 30-min cell incubation period [196]. Thus, it is necessary to establish ocular safety for each CS-based DDS and examine the degradation pathways and byproducts encountered, as well as determine whether they represent hazards to ocular tissues. Chronic delivery of therapeutic drug concentrations to the posterior eye segment is anticipated to necessitate the use of chitosan nanoparticles in high dosages in PSEDs. Consequently, providing both immediate and long-term safety at appropriate concentrations of CS is among the most crucial concerns that must be resolved. The ability to show reproducible production of the polymer is an important challenge for the clinical translation of CS-based devices. Chitosan is actually a mixture of many copolymers with varying residue ratios, rather than a single distinct molecule [197]. Therefore, it is critical to have a quality-controlled and well-defined chitosan grade, as variation in chitosan’s physicochemical properties (molecular weight, degree of deacetylation, etc.) affects not only activity but also mucoadhesion, permeation, biodegradability, and in vivo performance.

## Conclusion

Vision impairment is a significant public concern on a global scale, with profound implications for both developed and developing societies. The administration of these treatments, particularly to the posterior eye tissues, continues to be extremely challenging, despite the successful development of several promising therapeutic options for ocular diseases. Mucoadhesion is currently a non-invasive delivery method that has garnered significant interest. This is because it increases precorneal residence time and decreases precorneal clearance. By increasing the concentration gradient across ocular tissues, mucoadhesion facilitates the absorption of drugs from the conjunctiva and cornea to the posterior eye tissues after topical application. Among all the nanocarriers created for treating diabetic retinopathy, chitosan-based nanocarriers have attracted a lot of interest recently. These have numerous properties including ease of formulation, increased drug entrapment, and improved penetration and stability. Moreover, they are inexpensive, simple to use, and biologically beneficial. Researchers have shown that when anticancer treatment is administered via cubosomes, the likelihood of negative consequences to the patient is quite minimal. Nanoparticles of chitosan can treat diabetic retinopathy, therefore it can be utilized for enhancing diabetes patients’ quality of life and avoiding the unpleasant side effects of existing medicines.

## Data Availability

No datasets were generated or analysed during the current study.
